# Some distance measures for pythagorean cubic fuzzy sets: Application selection in optimal treatment for depression and anxiety

**DOI:** 10.1016/j.mex.2024.102678

**Published:** 2024-04-05

**Authors:** Muhammad Rahim, Fazli Amin, Kamal Shah, Thabet Abdeljawad, Sadique Ahmad

**Affiliations:** a Department of Mathematics and Statistics, Hazara University, Mansehra 21300, KP, Pakistan; b Department of Mathematics and Sciences, Prince Sultan University, Riyadh 11586, Saudi Arabia; c EIAS Data Science Lab, College of Computer and Information Sciences, Prince Sultan University, Riyadh, 11586, Saudi Arabia

**Keywords:** Generalized distance measures for Pythagorean cubic fuzzy sets, Pythagorean cubic fuzzy sets, Multi-criteria decision-making, Distance measures, Depression and anxiety

## Abstract

Pythagorean cubic fuzzy sets represent an advancement beyond conventional interval-valued Pythagorean sets, integrating the principles of Pythagorean fuzzy sets and interval-valued Pythagorean fuzzy sets. Given the critical significance of distance measures in real-world decision-making and pattern recognition tasks, it is noteworthy that there exists a notable gap in the literature regarding distance measures specifically tailored for Pythagorean cubic fuzzy sets. The objectives of this paper are:

• To define novel generalized distance measures between Pythagorean cubic fuzzy sets (PCFSs) to tackle intricate decision-making challenges.
• These novel distance measures are undergoing testing on a real-world scenario concerning the management of anxiety and depression to evaluate their effectiveness and practical application.
• We have illustrated the boundedness and nonlinear characteristics inherent in these distance measures.

In addition, we conduct comparative analyses with existing approaches to validate the proposed methodology, thereby providing insights into its advantages and potential applications.

## Graphical abstract

**Figure:**
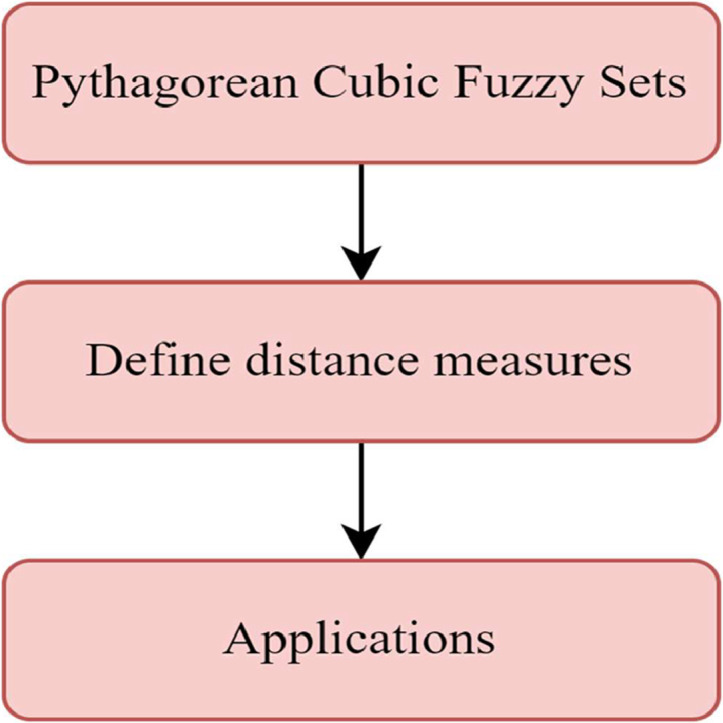

Specifications table

**Table:** 

Subject area:	Mathematics and Statistics
More specific subject area:	distance measures between Pythagorean cubic fuzzy sets
Name of your method:	generalized distance measures for Pythagorean cubic fuzzy sets
Name and reference of original method:	*Nill*
Resource availability:	*Nill*

**Method details**

## Introduction

The mental and emotional health of an individual may be severely impacted by depression and anxiety, therefore getting help is essential. The uniqueness of every individual's encounter with depression and anxiety makes treatment challenging. Moreover, sadness and anxiety do not share a single therapy. Drugs, therapy, or a hybrid of the two may be used in treatment. When designing a treatment plan for an individual, it is crucial to take into consideration their specific symptoms, medical history, preferences, resources, and therapy alternatives. To analyze and categorize options in light of many, often conflicting criteria, multi-criteria decision-making (MCDM) is employed [1], [2], [3]. It is often employed when more conventional approaches to evaluating decisions, including cost-benefit evaluation or net current value, could prove to be applicable or enough. Methods from MCDM can be utilized in several settings, including when deciding which investment opportunity to pursue, where to locate a new facility, or which choice will have the least impact on the environment. This highlights the need for methods and techniques that can effectively handle decision-making under uncertainty and ambiguity and help decision-makers to make informed decisions despite the limitations and challenges of real-world situations.

In 1965, Zadeh [4] introduced the concept of fuzzy sets (FS) to handle uncertainty and undetermined information in the decision-making process. The membership function of FS is defined between 0 and 1, which allows for a more flexible representation of information and makes it easier to handle uncertain and imprecise data. Atanassov [5] introduced the intuitionistic fuzzy set (IFS) in 1986. Since IFS is described by membership degree (MD) and non-membership degree (NMD). Based on IFS several studies are introduced see [6], [7], [8] and [9]. However, there may be cases when the sum of the MD and NMD provided by the decision maker is exceeds 1. To address these challenges, Yager [10] proposed the PFS with limitation that the square sum of its MD and NMD be less than or equal to 1. Different scholars have contributed attention to this issue under the PFS information. Yager [11], for instance, suggested a series of aggregation operators for PFS. The connection between Pythagorean MDs and complex numbers was investigated by Yager and Abbasov [12]. They developed the PF weighted geometric average operator based on this relationship. Zhang and Xu [13] introduced an enhanced approach for order preference by similarity to an ideal solution to address MCDM problems using PFS. Peng and Yang [14] defined two operational laws as division and subtraction and their properties. They also examined some possessions such as boundedness, monotonicity and idempotency of Pythagorean fuzzy aggregation operators. However, due to a lack of evidence in many decision-making difficulties, it may be challenging for decision-makers to properly assess their evaluations with a specific value. To assess such types of problems, Zhang [15] introduced IVPFSs which provide a more flexible platform for decision-makers.

Distance measure is a crucial concept within the field of FS theory and has been widely used in different fields. It plays an important role in these fields, as it allows for the quantification of the similarity or dissimilarity between fuzzy sets, which in turn is useful for making decisions, identifying patterns, training machine learning models, and forecasting market trends, among other applications [16], [17], [18], [19] and [20]. It has intrigued several researchers and they have delved deeper into it through further investigations. For example, Grzegorzewski [21] proposed Several Hausdorff distance-based strategies for comparing interval-valued fuzzy sets with intuitionistic fuzzy sets. A geometrical description of an intuitionistic fuzzy set and distances among intuitionistic fuzzy sets were provided by Szmidt and Kacprzyk [22]. For example, Xu and Chen [23] presented distance and similarity metrics for interval-valued intuitionistic fuzzy sets. Zeng and Guo [24] established normalized distance, similarity measure, and entropy measure. Talukdar and Dutta [25] introduced a set of distance measures for cubic Pythagorean fuzzy sets (CPFSs) and demonstrated their use in medical decision-making.

The cubic set [26] demonstrates its effectiveness in addressing potential disagreements that may arise from agreed-upon interval values, and conversely. Furthermore, it can incorporate two distinct time frames within a single Cubic Set (CS), enhancing its versatility in handling intricate scenarios. Within this framework, the analysis involves assessing the level of agreement or disagreement corresponding to the truth interval. Consider the following scenario: a supervisor is tasked with evaluating a subordinate's work. The subordinate presents a report indicating completion of 40 to 50% of the assigned tasks. After the supervisor's review, it is determined that there is a 20% discrepancy regarding the completion status. In such a scenario, a CS is expressed as an R-order, represented as ([0.40,0.50],0.20). Conversely, if the supervisor concurs with the subordinate's assessment of the completed tasks by 30%, a P-order CS is established as ([0.50,0.50],0.30). Consequently, through broadening the scope of the membership interval and incorporating a corresponding fuzzy set membership value, this framework enhances the accuracy level. The current approach relying on CSs fails to account for the NMD in conjunction with the MD, thereby restricting its practicality. To overcome this drawback, Abdullah [27] introduced the concept of intuitionistic cubic fuzzy sets (ICFS), amalgamating intuitionistic fuzzy sets (IFSs) and interval-valued intuitionistic fuzzy sets (IVIFSs) to efficiently manage fuzzy information. For instance, in a scenario where a supervisor evaluates a subordinate's work, suppose the subordinate submits a self-assessed report stating completion of 40−50% of assigned tasks while acknowledging non-completion of 30−40% of the work. After reviewing the report, an R-order CIFS could be described as (〈[0.40,0.50];20〉,〈[0.30,0.40];0.10〉) if the supervisor disagrees with 20% of the completed work and agrees with 10% of the incomplete work. Conversely, a P-order ICFS might be expressed as (〈[0.40,0.50];0.30〉,〈[0.30,0.40];0.10〉) if the supervisor concurs with 20% of the work done and disagrees with 30% of the uncompleted tasks according to the subordinate's assessment. In general, a ICFS can be expressed as $I=(\langle \left[\varphi_{I}^{L},\;\varphi_{I}^{U}\right];\lambda_{I}\rangle ,\;\langle \left[\psi_{I}^{L},\psi_{I}^{U}\right];\;\tau_{I}\rangle )$, where $\left[\varphi_{I}^{L},\;\varphi_{I}^{U}\right]$ and $\left[\psi_{I}^{L},\psi_{I}^{U}\right]$ are interval-valued fuzzy numbers satisfy the condition that $\varphi_{I}^{U}+\psi_{I}^{U}\leq 1$, while $\lambda_{I}$ and $\tau_{I}$ are fuzzy numbers such that $\lambda_{I}$,$\tau_{I}\in \left[0,1\right]$ and $\lambda_{I}+\tau_{I}\leq 1$.

The outlined requirements may not always be met in real-world scenarios. For instance, consider the set (〈[0.70,0.80];60〉,〈[0.40,0.50];0.70〉). Observing that 0.80+0.5>1 and 0.60+0.70>1, such information cannot be adequately accommodated within the ICFS framework. To address these limitations, Kahn et al. [28,29] introduced the concept of CPFSs. In general, a CPFS can be represented as $P=(\langle \left[\varphi_{P}^{L},\;\varphi_{P}^{U}\right];\lambda_{P}\rangle ,\;\langle \left[\psi_{P}^{L},\psi_{P}^{U}\right];\;\tau_{P}\rangle )$, where $\left[\varphi_{P}^{L},\;\varphi_{P}^{U}\right]$ and $\left[\psi_{P}^{L},\psi_{P}^{U}\right]$ are interval-valued fuzzy numbers satisfy the condition that ${\left(\varphi_{P}^{U}\right)}^{2}+{\left(\psi_{P}^{U}\right)}^{2}\leq 1$, while $\lambda_{P}$ and $\tau_{P}$ are fuzzy numbers such that $\lambda_{P}$,$\tau_{P}\in \left[0,1\right]$ and ${\left(\lambda_{I}\right)}^{2}+{\left(\tau_{I}\right)}^{2}\leq 1$. For handling intricate and nuanced fuzzy information, CPFSs offer a broader framework, facilitating more comprehensive modeling and analysis.

### Motivation and novelty of study

PCFSs are the most recent innovation in FS theory, and they provide decision-makers with a flexible framework for dealing with the uncertainty and imprecision of the available data. Despite its importance, studies on techniques for pinpointing the distance between two PFSs are in their infancy. PCFs excel in circumstances where judgment calls must be made, since their extra cubic element allows for a more nuanced comprehension of ambiguous and incorrect information. PCFSs are innovative because of the clarity and adaptability with which they may communicate important that is otherwise unclear. For this reason, PCFSs are particularly useful in decision-making applications assuming ambiguous or inaccurate information.

Unfortunately, the lack of research on distance measurements between two PCFSs represents an important obstacle to their wider acceptation in decision-making circumstances. Without such measures, decision-makers may struggle to compare and evaluate different sets, limiting the effectiveness of PCFSs as a tool for managing uncertainty and imprecision. Therefore, further research in this area is needed to unlock the full potential of PCFSs and enable their more widespread use in practical applications. To address the existing research gap, our proposal involves the development of new distance measures for PCFSs. Therefore, this study suggests the use of Euclidean distance, normalized Hamming distance, Hamming distance, and normalized weighted Euclidean distance as measures of similarity between two PCFSs. The contribution of the proposed study can be summarized as follow:

• Develop new distance measures between Pythagorean cubic fuzzy sets (PCFSs) to address complex decision-making issues.
• Novel distance measures have been tested in real-world scenarios for managing anxiety and depression to assess their effectiveness and practicality.
• We have demonstrated the boundedness and nonlinear features in these distance measures.

This study is structured in the following sections: Section 2 summaries the related work on anxiety and depression based on PCFSs. Section 3 provides a brief overview of PFSs, IVIFSs, CSs, and PCFSs. In Section 4, various distance measures for Pythagorean cubic fuzzy sets are introduced along with examples for better comprehension. Section 5 presents the application of Pythagorean cubic fuzzy sets and a case study showcasing the effectiveness of the proposed MCDM. Finally, Section 6 offers concluding remarks and brings the paper to a close.

## Related works

Studies have shown a formal description grade of membership analysis for the symptoms of some clinically defined psychiatric disorders such as panic disorder, social phobia, depression, simple phobia, generalized anxiety, agoraphobia, somatization and anxiety [30]. Depressive symptoms and depression diagnosis in a group of undergraduates were analyzed using a multivariate classification method [31]**.**

An intelligent support system based on fuzzy logic for the diagnosis of depression utilized fuzzy membership function and linguistic variables to capture the subjective nature of depression symptoms. By incorporating expert knowledge and patient-specific data, the system provides personalized recommendations for depression diagnosis [32]. The fuzzy logic-based approach incorporates fuzzy membership functions to evaluate depression symptoms and generate recommendations [33]. Fuzzy sets are applied to different fields for many purposes like to personalize the treatment of depression. One way to aid doctors in diagnosing depression is through a decision support system driven by neuro-fuzzy. In 2022 Liang et al. [34] used fuzzy qualitative comparative review to disclose the complex trajectory that contribute to depression in front-line nurses throughout the COVID-19 pandemic.

A system utilizing the Internet of Things is recommended for addressing this issue, wherein concepts related to depression are monitored and shared with healthcare professionals [35]. In a study conducted in 2023, Ramanna et al. [36] explored the potential effectiveness of cannabinoid-based medication as a therapeutic option for sleep disorders, anxiety, and depression amidst the COVID-19 pandemic. While various models and techniques have been developed for anxiety prediction, this study introduces a fuzzy logic-based classification model, specifically designed to capture the subjectivity and uncertainty inherent in anxiety assessment. The proposed system aims to deliver accurate predictions in the early stages of anxiety disorder [37].

In the broader field of PCFSs and their potential applications in mental health, such as depression and anxiety, there are gaps and challenges that need to be addressed. An important aspect is the need for more empirical studies to validate the effectiveness and reliability of different fuzzy logic models and systems in the treatment of mental health disorders. More precisely, there is a lack of rigorous research to evaluate the usefulness of PCFSs and their impact on optimal treatment results. Filling these gaps through additional research will help determine the potential benefits and practical viability of using PCFSs in the optimal treatment of depression and anxiety. Therefore, the focus of this study was optimal treatment for depression and anxiety based on PCFSs.

## Preliminaries

This section provides a brief overview of some fundamental concepts, such as PFSs, Cubic Set (CS), IVPFSs, and PCFSs, along with their properties. These concepts will be utilized in the subsequent sections of the paper.

### Pythagorean fuzzy sets

**Definition 1**[10]. A PFS P over an element t∈T is defined as:

(1) $$P=\left\{t,\;\langle \varphi_{P}\left(t\right),\;\psi_{P}\left(t\right)\rangle |t\in T\right\}$$

where $\varphi_{P}\left(t\right)$ and $\psi_{P}\left(t\right)$ represent the MD and NMD of an element t∈T such that

${\left(\varphi_{P}\left(t\right)\right)}^{2}+{\left(\psi_{P}\left(t\right)\right)}^{2}\leq 1$. The indeterminacy degree of t to P is defined as:

(2) $$\pi_{P}=\sqrt{1-{\left(\varphi_{P}\left(t\right)\right)}^{2}+{\left(\psi_{P}\left(t\right)\right)}^{2}}$$

for simplicity the pair $\langle \varphi_{P}\left(t\right),\;\psi_{P}\left(t\right)\rangle$ we represent as $P=(\varphi_{P},\psi_{P})$ and called Pythagrean fuzzy number (PFN).

**Definition 2**[13]. Let $P=(\varphi_{P},\psi_{P})$ be a PFN, then the score function over P is defined as:

(3) $$Sc\left(P\right)={\left(\varphi_{P}\right)}^{2}-{\left(\psi_{P}\right)}^{2}$$

where −1≤Sc(P)≤1, while the accuracy function over P is defined as:

(4) $$Ac\left(P\right)={\left(\varphi_{P}\right)}^{2}+{\left(\psi_{P}\right)}^{2}$$

where 0≤Sc(P)≤1.

Let $P_{1}$ and $P_{2}\;be$ two PFNs, then Yager and Abbasov [2] developed the following relationships:

1. If $Sc\left(P_{1}\right)<Sc\left(P_{2}\right)$ then $P_{1}<P_{2}$,
2. If $Sc\left(P_{1}\right)>Sc\left(P_{2}\right)$ then $P_{1}>P_{2}$,
3. If $Sc\left(P_{1}\right)=Sc\left(P_{2}\right)$ then
  • If $Ac\left(P_{1}\right)=Ac\left(P_{2}\right)$ then $P_{1}=P_{2}$,
  • If $Ac\left(P_{1}\right)<Ac\left(P_{2}\right)$ then $P_{1}<P_{2}$,
  • If $Ac\left(P_{1}\right)>Ac\left(P_{2}\right)$ then $P_{1}>P_{2}$.

This comparison approach for PFNs appears to be an extension of the comparison method for intuitionistic fuzzy numbers [2]. The findings of the two comparison procedures mentioned above for two PFNs might differ. Yager [24] also recommended using the weighted average aggregation operation to combine PFNs.

**Definition 3**[10]. Let $P_{1},\;P_{2},\ldots ,\;P_{n}$ be a family of PFNs and each $P_{i}=\left(\varphi_{i},\psi_{i}\right)$ be linked with weight $\vartheta_{i}$ such that $\vartheta_{i}>0$ where $\vartheta_{i}\in \left[0,\;1\right]$ and $\sum_{i=1}^{n}\vartheta_{i}=1$. Then the Pythagorean fuzzy weighted average (PFWA) operator is defined as follows:

(5) $$PFWA\left(P_{1},P_{2},\ldots ,P_{n}\right)=\left(\sum_{i=1}^{n}\vartheta_{i}\varphi_{i},\sum_{i=1}^{n}\vartheta_{i}\psi_{i}\right)$$

According to Zhang and Xu [7], the distance between two PFNs is as follows:

**Definition 4**[13]. Let $P_{1}$ and $P_{2}$ be two PFNs, the distance between $P_{1}$ and $P_{2}$ is defined as:

(6) $$\delta \left(P_{1},P_{2}\right)=\frac{1}{2}\left(\left|{\left(\varphi_{1}\right)}^{2}-{\left(\varphi_{2}\right)}^{2}\right|+\left|{\left(\psi_{1}\right)}^{2}-{\left(\psi_{2}\right)}^{2}\right|+\left|{\left(\pi_{1}\right)}^{2}-{\left(\pi_{2}\right)}^{2}\right|\right)$$

### Cubic set

**Definition 5** (Jun et al., 2012). Let T be a universal set. A CS over an element t∈T is presented as:

(7) $$C=\left\{t,\;{\tilde{\varphi }}_{C}\left(t\right),\;\lambda_{C}\left(t\right)|t\in T\right\}$$

where ${\tilde{\varphi }}_{C}\left(t\right)=\left[\varphi_{C}^{L}\left(t\right),\varphi_{C}^{U}\left(t\right)\right]\subset \left[0,1\right]$ is an interval-valued fuzzy set (IVFS) and $\lambda_{C}\left(t\right)\in \left[0,1\right]$ is a fuzzy set (FS) in set T**.**

### Interval-valued pythagorean fuzzy sets

**Definition 8**[15]. Let T be a finite set. An interval-valued Pythagorean fuzzy set (IVPFS) $\tilde{P}$ is defined as:

(8) $$\tilde{P}=\left\{t,\left[\varphi_{\tilde{P}}^{L}\left(t\right),\varphi_{\tilde{P}}^{U}\left(t\right)\right],\left[\psi_{\tilde{P}}^{L}\left(t\right),\psi_{\tilde{P}}^{U}\left(t\right)\right]|t\in T\right\}$$

where $\left[\varphi_{\tilde{P}}^{L},\varphi_{\tilde{P}}^{U}\right]\subset \left[0,1\right]$, $\left[\psi_{\tilde{P}}^{L},\psi_{\tilde{P}}^{U}\right]\subset \left[0,1\right]$ are the interval numbers, such that $\varphi_{\tilde{P}}^{L}\leq \varphi_{\tilde{P}}^{U}$, $\psi_{\tilde{P}}^{L}\leq \psi_{\tilde{P}}^{U}$ and ${\left(\varphi_{\tilde{P}}^{U}\right)}^{2}+{\left(\psi_{\tilde{P}}^{U}\right)}^{2}\leq 1$.

Let $\pi_{\tilde{P}}\left(t\right)=\left[\pi_{\tilde{P}}^{L}\left(t\right),\pi_{\tilde{P}}^{U}\right]$ for all t∈T, then it is called the IVPF index of t to T, where $\pi_{\tilde{P}}^{L}\left(x\right)=\sqrt{1-{\left(\varphi_{\tilde{P}}^{U}\left(t\right)\right)}^{2}-{\left(\psi_{\tilde{P}}^{U}\left(t\right)\right)}^{2}}$ and $\pi_{\tilde{P}}^{L}\left(x\right)=\sqrt{1-{\left(\varphi_{\tilde{P}}^{L}\left(t\right)\right)}^{2}-{\left(\psi_{\tilde{P}}^{L}\left(t\right)\right)}^{2}}$

**Property 1.** Let $\tilde{P}=\left(\left[\varphi_{\tilde{P}}^{L},\varphi_{\tilde{P}}^{U}\right],\left[\psi_{\tilde{P}}^{L},\psi_{\tilde{P}}^{U}\right]\right)$ be an interval-valued Pythagorean fuzzy number (IVPFN) then the following relations are satisfied.

1. If $\varphi_{\tilde{P}}^{L}=\varphi_{\tilde{P}}^{U}$ and $\psi_{\tilde{P}}^{L}=\psi_{\tilde{P}}^{U}$, then an IVPFN set reduces to PFN.
2. If $\varphi_{\tilde{P}}^{U}+\psi_{\tilde{P}}^{U}\leq 1$, then an IVPFN reduces to an interval-valued intuitionistic fuzzy number [27].

**Definition 9**[15]. Let $\tilde{P}=\left(\left[\varphi_{\tilde{P}}^{L},\varphi_{\tilde{P}}^{U}\right],\left[\psi_{\tilde{P}}^{L},\psi_{\tilde{P}}^{U}\right]\right)$ are the IVPFN, then the score function of $\tilde{P}$ is defined as:

(9) $$Sc\left(\tilde{P}\right)=\frac{1}{2}\left({\left(\varphi_{\tilde{P}}^{L}\right)}^{2}+{\left(\varphi_{\tilde{P}}^{U}\right)}^{2}-{\left(\psi_{\tilde{P}}^{L}\right)}^{2}-{\left(\psi_{\tilde{P}}^{U}\right)}^{2}\right)$$

where $0\leq Sc(\tilde{P})\leq 1$.

**Definition 10**[15]. Let $\tilde{P}=\left(\left[\varphi_{\tilde{P}}^{L},\varphi_{\tilde{P}}^{U}\right],\left[\psi_{\tilde{P}}^{L},\psi_{\tilde{P}}^{U}\right]\right)$ be an IVPFN then the accuracy function of $\tilde{P}$ is defined as:

(10) $$Ac\left(\tilde{P}\right)=\frac{1}{2}\left({\left(\varphi_{\tilde{P}}^{L}\right)}^{2}+{\left(\varphi_{\tilde{P}}^{U}\right)}^{2}+{\left(\psi_{\tilde{P}}^{L}\right)}^{2}+{\left(\psi_{\tilde{P}}^{U}\right)}^{2}\right)$$

where $-1\leq Sc(\tilde{P})\leq 1$.

**Definition 11**[15]. Let ${\tilde{P}}_{i}\;\left(i=1,2\right)$ be two IVPFNs. The distance measure between ${\tilde{P}}_{1}$and ${\tilde{P}}_{2}$ is represented as follows:

(11) $$\begin{array}{ccc} \delta \left({\tilde{P}}_{1},{\tilde{P}}_{2}\right) & = & \frac{1}{6}(\left|{\left(\varphi_{{\tilde{P}}_{1}}^{L}\right)}^{2}-{\left(\varphi_{{\tilde{P}}_{2}}^{L}\right)}^{2}\right|+\left|{\left(\varphi_{{\tilde{P}}_{1}}^{U}\right)}^{2}-{\left(\varphi_{{\tilde{P}}_{2}}^{U}\right)}^{2}\right|+\left|{\left(\psi_{{\tilde{P}}_{1}}^{L}\right)}^{2}-{\left(\psi_{{\tilde{P}}_{2}}^{L}\right)}^{2}\right|\;+\left|{\left(\psi_{{\tilde{P}}_{1}}^{U}\right)}^{2}-{\left(\varphi_{{\tilde{P}}_{2}}^{U}\right)}^{2}\right|\\ & & +\left|{\left(\pi_{{\tilde{P}}_{1}}\right)}^{2}-{\left(\pi_{{\tilde{P}}_{1}}\right)}^{2}\right|+\left|{\left(\xi_{{\tilde{P}}_{1}}\right)}^{2}-{\left(\xi_{{\tilde{P}}_{1}}\right)}^{2}\right|\;) \end{array}$$

where $\left[\pi_{{\tilde{P}}_{1}},\xi_{{\tilde{P}}_{1}}\right]=\left(\left[\sqrt{1-{\left(\varphi_{{\tilde{P}}_{1}}^{L}\right)}^{2}-{\left(\psi_{{\tilde{P}}_{1}}^{L}\right)}^{2}},\sqrt{1-{\left(\varphi_{{\tilde{P}}_{1}}^{U}\right)}^{2}-{\left(\psi_{{\tilde{P}}_{1}}^{U}\right)}^{2}}\right]\right)$ and $\left[\pi_{{\tilde{P}}_{2}},\xi_{{\tilde{P}}_{2}}\right]=\left(\left[\sqrt{1-{\left(\varphi_{{\tilde{P}}_{2}}^{L}\right)}^{2}-{\left(\psi_{{\tilde{P}}_{2}}^{L}\right)}^{2}},\sqrt{1-{\left(\varphi_{{\tilde{P}}_{2}}^{U}\right)}^{2}-{\left(\psi_{{\tilde{P}}_{2}}^{U}\right)}^{2}}\right]\right)$.

### Pythagorean cubic fuzzy sets and their properties

**Definition 12** (Khan et al., 2019). Let T be a non-empty finite set. A PCFS $P_{C}$ over an element t∈T is defined as:

(12) $$P_{C}=\left\{t,\;\varphi_{P_{C}}\left(t\right),\psi_{P_{C}}\left(t\right)|t\in T\right\}$$

where $\varphi_{P_{C}}\left(t\right)=\left(\left[\varphi_{P_{C}}^{L}\left(t\right),\varphi_{P_{C}}^{U}\left(t\right)\right];\lambda_{P_{C}}\left(t\right)\right)$ and $\psi_{P_{C}}\left(t\right)=\left(\left[\psi_{P_{C}}^{L}\left(t\right),\psi_{P_{C}}^{U}\left(t\right)\right];\tau_{P_{C}}\left(t\right)\right)$ are two cubic sets which characterizes the MD and NMD of $P_{C}$ such that ${\left(\varphi_{P_{C}}^{U}\left(t\right)\right)}^{2}+{\left(\psi_{P_{C}}^{U}\left(t\right)\right)}^{2}\leq 1$ and ${\left(\lambda_{P_{C}}\left(t\right)\right)}^{2}+{\left(\tau_{P_{C}}\left(t\right)\right)}^{2}\leq 1$.

**Definition 13** (Khan et al., 2019). Let $P_{C}=\left(\varphi_{P_{C}},\psi_{P_{C}}\right)$ be a PCFN, where $\varphi_{P_{C}}\left(t\right)=\left(\left[\varphi_{P_{C}}^{L},\varphi_{P_{C}}^{U}\right];\lambda_{P_{C}}\right)$ and $\psi_{P_{C}}\left(t\right)=\left(\left[\psi_{P_{C}}^{L},\psi_{P_{C}}^{U}\right];\tau_{P_{C}}\right)$. Then the score function of $P_{C}$ is defined as:

(13) $$Sc\left(P_{C}\right)={\left(\frac{\varphi_{P_{C}}^{L}+\varphi_{P_{C}}^{U}-\lambda_{P_{C}}}{3}\right)}^{2}-{\left(\frac{\psi_{P_{C}}^{L}+\psi_{P_{C}}^{U}-\tau_{P_{C}}}{3}\right)}^{2}$$

where $-1\leq Sc(P_{C})\leq 1$.

**Definition 14**[28]. Let $P_{C_{1}}$ and $P_{C_{2}}$ be two PCFNs, then

1. If $Sc\left(P_{C_{1}}\right)<Sc\left(P_{C_{2}}\right)$ then $P_{C_{1}}<P_{C_{2}}$,
2. If $Sc\left(P_{C_{1}}\right)>Sc\left(P_{C_{2}}\right)$ then $P_{C_{1}}>P_{C_{2}}$,
3. If $Sc\left(P_{C_{1}}\right)=Sc\left(P_{C_{2}}\right)$ then $P_{C_{1}}∼P_{C_{2}}$.
  (14) $$Ac\left(P_{C}\right)={\left(\frac{\varphi_{P_{C}}^{L}+\varphi_{P_{C}}^{U}+\lambda_{P_{C}}}{3}\right)}^{2}+{\left(\frac{\psi_{P_{C}}^{L}+\psi_{P_{C}}^{U}+\tau_{P_{C}}}{3}\right)}^{2}$$

where $0\leq Ac(P_{C})\leq 1$.

**Definition 15**[28]. Let $P_{C}=\left(\varphi_{P_{C}},\psi_{P_{C}}\right)$ be a PCFN, where $\varphi_{P_{C}}\left(t\right)=\left(\left[\varphi_{P_{C}}^{L},\varphi_{P_{C}}^{U}\right];\lambda_{P_{C}}\right)$ and $\psi_{P_{C}}\left(t\right)=\left(\left[\psi_{P_{C}}^{L},\psi_{P_{C}}^{U}\right];\tau_{P_{C}}\right)$. Then the accuracy function of $P_{C}$ is defined as:

**Definition 16**[28]. Let $P_{C_{1}}$ and $P_{C_{2}}$ be two PCFNs and, then

1. If $Ac\left(P_{C_{1}}\right)<Ac\left(P_{C_{2}}\right)$ then $P_{C_{1}}<P_{C_{2}}$,
2. If $Ac\left(P_{C_{1}}\right)>Ac\left(P_{C_{2}}\right)$ then $P_{C_{1}}>P_{C_{2}}$,
3. If $Ac\left(P_{C_{1}}\right)=Ac\left(P_{C_{2}}\right)$ then $P_{C_{1}}∼P_{C_{2}}$.

## Novel distance measures for PCFNs and PCFSs

The main attraction of PCFN is that it is defined with six parameters: $\varphi_{P_{C}}^{L}$, $\varphi_{P_{C}}^{U}$, $\psi_{P_{C}}^{L}$, $\psi_{P_{C}}^{U}$, $\lambda_{P_{C}}$ and $\tau_{P_{C}}$. We propose the following distance measures for PCFNs, which takes into consideration the differences in the parameter values.

**Definition 17.** Let $P_{C}=\left(\langle \left[\varphi_{C_{P}}^{L},\;\varphi_{P_{C}}^{U}\right];\lambda_{P_{C}}\rangle ,\;\langle \left[\psi_{P_{C}}^{L},\psi_{P_{C}}^{U}\right];\;\tau_{P_{C}}\rangle \right)\;(i=1,2$) be the collections of PCFNs. Then $\pi_{P_{C}}\left(t\right)=\left(\left[\pi_{P_{C}}^{L}\left(t\right),\pi_{P_{C}}^{U}\left(t\right)\right];\pi_{P_{C}}\left(t\right)\right)$ is said to be a PCF index of t∈T where

(15) $$\pi_{P_{C}}^{L}\left(t\right)=\sqrt{1-{\left(\varphi_{P_{C}}^{U}\right)}^{2}-{\left(\psi_{P_{C}}^{U}\right)}^{2}}$$

(16) $$\pi_{P_{C}}^{U}\left(t\right)=\sqrt{1-{\left(\varphi_{P_{C}}^{L}\right)}^{2}-{\left(\psi_{P_{C}}^{L}\right)}^{2}}$$

(17) $$\pi_{P_{C}}\left(t\right)=\sqrt{1-{\left(\lambda_{P_{C}}\right)}^{2}-{\left(\tau_{P_{C}}\right)}^{2}}$$

For simplicity, we call $\left(\varphi_{P_{C}},\psi_{P_{C}}\right)$ a PCFN denoted by $P_{C}=\left(\varphi_{P_{C}},\psi_{P_{C}}\right)$. Here $\varphi_{P_{C}}$ represents the degree of satisfaction of t in T and the degree of rejection or against of the t in T.

### Hamming distance

**Definition 17.** Let $P_{C_{1}}$ and $P_{C_{2}}$ be two PCFNs where

$P_{C_{1}}=\left(\left[\varphi_{P_{C_{1}}}^{L},\varphi_{P_{C_{1}}}^{U}\right];\lambda_{P_{C_{1}}},\left[\psi_{P_{C_{1}}}^{L},\psi_{P_{C_{1}}}^{U}\right];\tau_{P_{C_{1}}}\right)$ and $P_{C_{1}}=\left(\left[\varphi_{P_{C_{2}}}^{L},\varphi_{P_{C_{2}}}^{U}\right];\lambda_{P_{C_{2}}},\left[\psi_{P_{C_{2}}}^{L},\psi_{P_{C_{2}}}^{U}\right];\tau_{P_{C_{2}}}\right)$, then normalized Hamming distance (NHD) measure between $P_{C_{1}}$and $P_{C_{2}}$ is presented as follows:

(18) $$\begin{array}{ccc} \delta_{H} & = & \frac{1}{6}(\left|{\left(\varphi_{P_{C_{1}}}^{L}\right)}^{2}-{\left(\varphi_{P_{C_{2}}}^{L}\right)}^{2}\right|+\left|{\left(\varphi_{P_{C_{1}}}^{U}\right)}^{2}-{\left(\varphi_{P_{C_{2}}}^{U}\right)}^{2}\right|+\left|{\left(\psi_{P_{C_{1}}}^{L}\right)}^{2}-{\left(\psi_{P_{C_{2}}}^{L}\right)}^{2}\right|+\;\left|{\left(\psi_{P_{C_{1}}}^{U}\right)}^{2}-{\left(\psi_{P_{C_{1}}}^{U}\right)}^{2}\right|\\ & & +\left|{\left(\lambda_{P_{C_{1}}}\right)}^{2}-{\left(\lambda_{P_{C_{2}}}\right)}^{2}\right|+\left|{\left(\tau_{P_{C_{1}}}\right)}^{2}-{\left(\tau_{P_{C_{2}}}\right)}^{2}\right|+\left|{\left(\pi_{P_{C_{1}}}^{L}\right)}^{2}-{\left(\pi_{P_{C_{2}}}^{L}\right)}^{2}\right|+\left|{\left(\pi_{P_{C_{1}}}^{U}\right)}^{2}-{\left(\pi_{P_{C_{2}}}^{U}\right)}^{2}\right|+\left|{\left(\pi_{P_{C_{1}}}\right)}^{2}-{\left(\pi_{P_{C_{2}}}\right)}^{2}\right|\;) \end{array}$$

The weighted Hamming distance measure can be calculated as

(19) $$\begin{array}{ccc} \delta_{\xi H} & = & \frac{1}{6}\xi_{i}(\left|{\left(\varphi_{P_{C_{1}}}^{L}\right)}^{2}-{\left(\varphi_{P_{C_{2}}}^{L}\right)}^{2}\right|+\left|{\left(\varphi_{P_{C_{1}}}^{U}\right)}^{2}-{\left(\varphi_{P_{C_{2}}}^{U}\right)}^{2}\right|+\left|{\left(\psi_{P_{C_{1}}}^{L}\right)}^{2}-{\left(\psi_{P_{C_{2}}}^{L}\right)}^{2}\right|+\;\left|{\left(\psi_{P_{C_{1}}}^{U}\right)}^{2}-{\left(\psi_{P_{C_{1}}}^{U}\right)}^{2}\right|+\left|{\left(\lambda_{P_{C_{1}}}\right)}^{2}-{\left(\lambda_{P_{C_{2}}}\right)}^{2}\right|\\ & & +\left|{\left(\tau_{P_{C_{1}}}\right)}^{2}-{\left(\tau_{P_{C_{2}}}\right)}^{2}\right|+\;+\left|{\left(\pi_{P_{C_{1}}}^{L}\right)}^{2}-{\left(\pi_{P_{C_{2}}}^{L}\right)}^{2}\right|+\left|{\left(\pi_{P_{C_{1}}}^{U}\right)}^{2}-{\left(\pi_{P_{C_{2}}}^{U}\right)}^{2}\right|+\left|{\left(\pi_{P_{C_{1}}}\right)}^{2}-{\left(\pi_{P_{C_{2}}}\right)}^{2}\right|\;) \end{array}$$

where $\sum_{i=1}^{6}\xi_{i}=1$.

**Example 1.** Let $P_{C_{1}}=\left(\langle [0.2,0.7];0.5\rangle ,\langle [0.3,0.6];0.6\rangle \right)$ and $P_{C_{2}}=\left(\langle [0.4,0.8];0.5\rangle ,\langle [0.3,0.5];0.4\rangle \right)$. then NHD between $P_{C_{1}}$ and $P_{C_{2}}$ can be calculated as:

$$\pi_{P_{C_{1}}}^{L}\left(t\right)=\sqrt{1-{\left(\varphi_{P_{C_{1}}}^{U}\right)}^{2}-{\left(\psi_{P_{C_{1}}}^{U}\right)}^{2}}=\sqrt{1-{\left(0.7\right)}^{2}-{\left(0.6\right)}^{2}}=0.3873$$

$$\pi_{P_{C_{2}}}^{L}\left(t\right)=\sqrt{1-{\left(\varphi_{P_{C_{2}}}^{U}\right)}^{2}-{\left(\psi_{P_{C_{2}}}^{U}\right)}^{2}}=\sqrt{1-{\left(0.8\right)}^{2}-{\left(0.5\right)}^{2}}=0.3317$$

$$\pi_{P_{C_{1}}}^{U}\left(t\right)=\sqrt{1-{\left(\varphi_{P_{C_{1}}}^{L}\right)}^{2}-{\left(\psi_{P_{C_{1}}}^{L}\right)}^{2}}=\sqrt{1-{\left(0.2\right)}^{2}-{\left(0.3\right)}^{2}}=0.9327$$

$$\pi_{P_{C_{2}}}^{U}\left(t\right)=\sqrt{1-{\left(\varphi_{P_{C_{2}}}^{L}\right)}^{2}-{\left(\psi_{P_{C_{2}}}^{L}\right)}^{2}}=\sqrt{1-{\left(0.4\right)}^{2}-{\left(0.3\right)}^{2}}=0.8660$$

$$\pi_{P_{C_{1}}}\left(t\right)=\sqrt{1-{\left(\lambda_{P_{C_{1}}}\right)}^{2}-{\left(\tau_{P_{C_{1}}}\right)}^{2}}=\sqrt{1-{\left(0.5\right)}^{2}-{\left(0.6\right)}^{2}}=0.6245$$

$$\pi_{P_{C_{2}}}\left(t\right)=\sqrt{1-{\left(\lambda_{P_{C_{2}}}\right)}^{2}-{\left(\tau_{P_{C_{2}}}\right)}^{2}}=\sqrt{1-{\left(0.5\right)}^{2}-{\left(0.4\right)}^{2}}=0.7681.$$

$$\begin{array}{ccc} \delta_{H} & = & \frac{1}{6}(\left|{\left(\varphi_{P_{C_{1}}}^{L}\right)}^{2}-{\left(\varphi_{P_{C_{2}}}^{L}\right)}^{2}\right|+\left|{\left(\varphi_{P_{C_{1}}}^{U}\right)}^{2}-{\left(\varphi_{P_{C_{2}}}^{U}\right)}^{2}\right|+\left|{\left(\psi_{P_{C_{1}}}^{L}\right)}^{2}-{\left(\psi_{P_{C_{2}}}^{L}\right)}^{2}\right|+\;\left|{\left(\psi_{P_{C_{1}}}^{U}\right)}^{2}-{\left(\psi_{P_{C_{1}}}^{U}\right)}^{2}\right|+\left|{\left(\lambda_{P_{C_{1}}}\right)}^{2}-{\left(\lambda_{P_{C_{2}}}\right)}^{2}\right|\\ & & +\left|{\left(\tau_{P_{C_{1}}}\right)}^{2}-{\left(\tau_{P_{C_{2}}}\right)}^{2}\right|+\;+\left|{\left(\pi_{P_{C_{1}}}^{L}\right)}^{2}-{\left(\pi_{P_{C_{2}}}^{L}\right)}^{2}\right|+\left|{\left(\pi_{P_{C_{1}}}^{U}\right)}^{2}-{\left(\pi_{P_{C_{2}}}^{U}\right)}^{2}\right|+\left|{\left(\pi_{P_{C_{1}}}\right)}^{2}-{\left(\pi_{P_{C_{2}}}\right)}^{2}\right|\;) \end{array}$$

$$\begin{array}{ccc} & = & \frac{1}{6}(\left|{\left(0.2\right)}^{2}-{\left(0.4\right)}^{2}\right|+\left|{\left(0.7\right)}^{2}-{\left(0.8\right)}^{2}\right|+\left|{\left(0.3\right)}^{2}-{\left(0.3\right)}^{2}\right|+\;\left|{\left(0.6\right)}^{2}-{\left(0.5\right)}^{2}\right|+\left|{\left(0.5\right)}^{2}-{\left(0.5\right)}^{2}\right|\\ & & +\;\left|{\left(0.6\right)}^{2}-{\left(0.4\right)}^{2}\right|+\;+\left|{\left(0.3873\right)}^{2}-{\left(0.3317\right)}^{2}\right|+\left|{\left(0.9327\right)}^{2}-{\left(0.8660\right)}^{2}\right|+\left|{\left(0.6245\right)}^{2}-{\left(0.7681\right)}^{2}\right|\;) \end{array}$$

=0.2900

### Euclidean distance (ED)

**Definition 18.** Let $P_{C_{1}}$ and $P_{C_{2}}$ be any two PCFNs. The normalized Euclidean distance (NED) between $P_{C_{1}}$ and $P_{C_{2}}$ can be defined as follow:

(20) $$\delta_{E}\left(P_{C_{1}},P_{C_{2}}\right)=\sqrt{\frac{1}{6}\left(\begin{array}{c} {\left|{\left(\varphi_{P_{C_{1}}}^{L}\right)}^{2}-{\left(\varphi_{P_{C_{2}}}^{L}\right)}^{2}\right|}^{2}+{\left|{\left(\varphi_{P_{C_{1}}}^{U}\right)}^{2}-{\left(\varphi_{P_{C_{2}}}^{U}\right)}^{2}\right|}^{2}+{\left|{\left(\psi_{P_{C_{1}}}^{L}\right)}^{2}-{\left(\psi_{P_{C_{2}}}^{L}\right)}^{2}\right|}^{2}\\ +\;{\left|{\left(\psi_{P_{C_{1}}}^{U}\right)}^{2}-{\left(\psi_{P_{C_{1}}}^{U}\right)}^{2}\right|}^{2}+{\left|{\left(\lambda_{P_{C_{1}}}\right)}^{2}-{\left(\lambda_{P_{C_{2}}}\right)}^{2}\right|}^{2}+{\left|{\left(\tau_{P_{C_{1}}}\right)}^{2}-{\left(\tau_{P_{C_{2}}}\right)}^{2}\right|}^{2}\\ +\;{\left|{\left(\pi_{P_{C_{1}}}^{L}\right)}^{2}-{\left(\pi_{P_{C_{2}}}^{L}\right)}^{2}\right|}^{2}+{\left|{\left(\pi_{P_{C_{1}}}^{U}\right)}^{2}-{\left(\pi_{P_{C_{2}}}^{U}\right)}^{2}\right|}^{2}+{\left|{\left(\pi_{P_{C_{1}}}\right)}^{2}-{\left(\pi_{P_{C_{2}}}\right)}^{2}\right|}^{2} \end{array}\right)}$$

The normalized generalized distance (NGD) between $P_{C_{1}}$ and $P_{C_{2}}$ is defined as:

(21) $$\delta_{G}\left(P_{C_{1}},P_{C_{2}}\right)={\left(\frac{1}{6}\left(\begin{array}{c} {\left|{\left(\varphi_{P_{C_{1}}}^{L}\right)}^{2}-{\left(\varphi_{P_{C_{2}}}^{L}\right)}^{2}\right|}^{\gamma }+{\left|{\left(\varphi_{P_{C_{1}}}^{U}\right)}^{2}-{\left(\varphi_{P_{C_{2}}}^{U}\right)}^{2}\right|}^{\gamma }+{\left|{\left(\psi_{P_{C_{1}}}^{L}\right)}^{2}-{\left(\psi_{P_{C_{2}}}^{L}\right)}^{2}\right|}^{\gamma }\\ +\;\;{\left|{\left(\psi_{P_{C_{1}}}^{U}\right)}^{2}-{\left(\psi_{P_{C_{1}}}^{U}\right)}^{2}\right|}^{\gamma }+{\left|{\left(\lambda_{P_{C_{1}}}\right)}^{2}-{\left(\lambda_{P_{C_{2}}}\right)}^{2}\right|}^{\gamma }+{\left|{\left(\tau_{P_{C_{1}}}\right)}^{2}-{\left(\tau_{P_{C_{2}}}\right)}^{2}\right|}^{\gamma }\\ \;+{\left|{\left(\pi_{P_{C_{1}}}^{L}\right)}^{2}-{\left(\pi_{P_{C_{2}}}^{L}\right)}^{2}\right|}^{\gamma }+{\left|{\left(\pi_{P_{C_{1}}}^{U}\right)}^{2}-{\left(\pi_{P_{C_{2}}}^{U}\right)}^{2}\right|}^{\gamma }+{\left|{\left(\pi_{P_{C_{1}}}\right)}^{2}-{\left(\pi_{P_{C_{2}}}\right)}^{2}\right|}^{\gamma } \end{array}\right)\right)}^{\frac{1}{\gamma }}$$

**Example 2.** Let $P_{C_{1}}=\left(\langle [0.3,0.4];0.6\rangle ,\langle [0.6,0.7];0.4\rangle \right)$ and $P_{C_{2}}=\left(\langle [0.4,0.5];0.5\rangle ,\langle [0.7,0.8];0.3\rangle \right)$. then NED between $P_{C_{1}}$ and $P_{C_{2}}$ can be calculated as:

$$\pi_{P_{C_{1}}}^{L}\left(t\right)=\sqrt{1-{\left(\varphi_{P_{C_{1}}}^{U}\right)}^{2}-{\left(\psi_{P_{C_{1}}}^{U}\right)}^{2}}=\sqrt{1-{\left(0.4\right)}^{2}-{\left(0.7\right)}^{2}}=0.5916$$

$$\pi_{P_{C_{2}}}^{L}\left(t\right)=\sqrt{1-{\left(\varphi_{P_{C_{2}}}^{U}\right)}^{2}-{\left(\psi_{P_{C_{2}}}^{U}\right)}^{2}}=\sqrt{1-{\left(0.5\right)}^{2}-{\left(0.8\right)}^{2}}=0.3317$$

$$\pi_{P_{C_{1}}}^{U}\left(t\right)=\sqrt{1-{\left(\varphi_{P_{C_{1}}}^{L}\right)}^{2}-{\left(\psi_{P_{C_{1}}}^{L}\right)}^{2}}=\sqrt{1-{\left(0.3\right)}^{2}-{\left(0.6\right)}^{2}}=0.7416$$

$$\pi_{P_{C_{2}}}^{U}\left(t\right)=\sqrt{1-{\left(\varphi_{P_{C_{2}}}^{L}\right)}^{2}-{\left(\psi_{P_{C_{2}}}^{L}\right)}^{2}}=\sqrt{1-{\left(0.4\right)}^{2}-{\left(0.7\right)}^{2}}=0.5916$$

$$\pi_{P_{C_{1}}}\left(t\right)=\sqrt{1-{\left(\lambda_{P_{C_{1}}}\right)}^{2}-{\left(\tau_{P_{C_{1}}}\right)}^{2}}=\sqrt{1-{\left(0.6\right)}^{2}-{\left(0.4\right)}^{2}}=0.6928$$

$$\pi_{P_{C_{2}}}\left(t\right)=\sqrt{1-{\left(\lambda_{P_{C_{2}}}\right)}^{2}-{\left(\tau_{P_{C_{2}}}\right)}^{2}}=\sqrt{1-{\left(0.5\right)}^{2}-{\left(0.3\right)}^{2}}=0.8124.$$

$$\delta_{E}\left(P_{C_{1}},P_{C_{2}}\right)=\sqrt{\frac{1}{6}\left(\begin{array}{c} {\left|{\left(\varphi_{P_{C_{1}}}^{L}\right)}^{2}-{\left(\varphi_{P_{C_{2}}}^{L}\right)}^{2}\right|}^{2}+{\left|{\left(\varphi_{P_{C_{1}}}^{U}\right)}^{2}-{\left(\varphi_{P_{C_{2}}}^{U}\right)}^{2}\right|}^{2}+{\left|{\left(\psi_{P_{C_{1}}}^{L}\right)}^{2}-{\left(\psi_{P_{C_{2}}}^{L}\right)}^{2}\right|}^{2}\\ +\;{\left|{\left(\psi_{P_{C_{1}}}^{U}\right)}^{2}-{\left(\psi_{P_{C_{1}}}^{U}\right)}^{2}\right|}^{2}+{\left|{\left(\lambda_{P_{C_{1}}}\right)}^{2}-{\left(\lambda_{P_{C_{2}}}\right)}^{2}\right|}^{2}+{\left|{\left(\tau_{P_{C_{1}}}\right)}^{2}-{\left(\tau_{P_{C_{2}}}\right)}^{2}\right|}^{2}\\ +\;{\left|{\left(\pi_{P_{C_{1}}}^{L}\right)}^{2}-{\left(\pi_{P_{C_{2}}}^{L}\right)}^{2}\right|}^{2}+{\left|{\left(\pi_{P_{C_{1}}}^{U}\right)}^{2}-{\left(\pi_{P_{C_{2}}}^{U}\right)}^{2}\right|}^{2}+{\left|{\left(\pi_{P_{C_{1}}}\right)}^{2}-{\left(\pi_{P_{C_{2}}}\right)}^{2}\right|}^{2} \end{array}\right)}$$

$$\begin{array}{ccc} \delta_{E}\left(P_{C_{1}},P_{C_{2}}\right) & = & \sqrt{\frac{1}{6}\left(\begin{array}{c} {\left|{\left(0.3\right)}^{2}-{\left(0.4\right)}^{2}\right|}^{2}+{\left|{\left(0.4\right)}^{2}-{\left(0.5\right)}^{2}\right|}^{2}+{\left|{\left(0.6\right)}^{2}-{\left(0.7\right)}^{2}\right|}^{2}\;\\ +\;{\left|{\left(0.7\right)}^{2}-{\left(0.8\right)}^{2}\right|}^{2}+{\left|{\left(0.6\right)}^{2}-{\left(0.5\right)}^{2}\right|}^{2}+{\left|{\left(0.4\right)}^{2}-{\left(0.3\right)}^{2}\right|}^{2}\;\\ +\;{\left|{\left(0.5916\right)}^{2}-{\left(0.3317\right)}^{2}\right|}^{2}+{\left|{\left(0.7416\right)}^{2}-{\left(0.5916\right)}^{2}\right|}^{2}\;\\ +\;{\left|{\left(0.6928\right)}^{2}-{\left(0.8124\right)}^{2}\right|}^{2}\; \end{array}\right)\;}\\ & = & 0.0744. \end{array}$$

**Definition 19.** Let $P_{C_{1}}$ and $P_{C_{2}}$ be any two PCFNs and ξ be the weight vector. The normalized weighted Euclidean distance (NWED) between $P_{C_{1}}$ and $P_{C_{2}}$ can be defined in the following way:

(22) $$\delta_{\xi E}\left(P_{C_{1}},P_{C_{2}}\right)=\sqrt{\frac{1}{6}\left(\begin{array}{c} \xi_{1}{\left|{\left(\varphi_{P_{C_{1}}}^{L}\right)}^{2}-{\left(\varphi_{P_{C_{2}}}^{L}\right)}^{2}\right|}^{2}+\xi_{2}{\left|{\left(\varphi_{P_{C_{1}}}^{U}\right)}^{2}-{\left(\varphi_{P_{C_{2}}}^{U}\right)}^{2}\right|}^{2}\\ +\;\xi_{3}{\left|{\left(\psi_{P_{C_{1}}}^{L}\right)}^{2}-{\left(\psi_{P_{C_{2}}}^{L}\right)}^{2}\right|}^{2}\;+\;\xi_{4}{\left|{\left(\psi_{P_{C_{1}}}^{U}\right)}^{2}-{\left(\psi_{P_{C_{1}}}^{U}\right)}^{2}\right|}^{2}\\ +\;\xi_{5}{\left|{\left(\lambda_{P_{C_{1}}}\right)}^{2}-{\left(\lambda_{P_{C_{2}}}\right)}^{2}\right|}^{2}+\xi_{6}{\left|{\left(\tau_{P_{C_{1}}}\right)}^{2}-{\left(\tau_{P_{C_{2}}}\right)}^{2}\right|}^{2}\\ +\;\xi_{7}{\left|{\left(\pi_{P_{C_{1}}}^{L}\right)}^{2}-{\left(\pi_{P_{C_{2}}}^{L}\right)}^{2}\right|}^{2}+\xi_{8}{\left|{\left(\pi_{P_{C_{1}}}^{U}\right)}^{2}-{\left(\pi_{P_{C_{2}}}^{U}\right)}^{2}\right|}^{2}\\ +\;\xi_{9}{\left|{\left(\pi_{P_{C_{1}}}\right)}^{2}-{\left(\pi_{P_{C_{2}}}\right)}^{2}\right|}^{2} \end{array}\right)}$$

The weighted NGD measure between $P_{C_{1}}$ and $P_{C_{2}}$ is defined as:

(23) $$\delta_{\xi G}\left(P_{C_{1}},P_{C_{2}}\right)={\left(\frac{1}{6}\left(\begin{array}{c} \xi_{1}{\left|{\left(\varphi_{P_{C_{1}}}^{L}\right)}^{2}-{\left(\varphi_{P_{C_{2}}}^{L}\right)}^{2}\right|}^{\gamma }+\xi_{2}{\left|{\left(\varphi_{P_{C_{1}}}^{U}\right)}^{2}-{\left(\varphi_{P_{C_{2}}}^{U}\right)}^{2}\right|}^{\gamma }+\xi_{3}{\left|{\left(\psi_{P_{C_{1}}}^{L}\right)}^{2}-{\left(\psi_{P_{C_{2}}}^{L}\right)}^{2}\right|}^{\gamma }\\ +\;\xi_{4}{\left|{\left(\psi_{P_{C_{1}}}^{U}\right)}^{2}-{\left(\psi_{P_{C_{1}}}^{U}\right)}^{2}\right|}^{\gamma }+\xi_{5}{\left|{\left(\lambda_{P_{C_{1}}}\right)}^{2}-{\left(\lambda_{P_{C_{2}}}\right)}^{2}\right|}^{\gamma }+\xi_{6}{\left|{\left(\tau_{P_{C_{1}}}\right)}^{2}-{\left(\tau_{P_{C_{2}}}\right)}^{2}\right|}^{\gamma }\\ +\xi_{7}{\left|{\left(\pi_{P_{C_{1}}}^{L}\right)}^{2}-{\left(\pi_{P_{C_{2}}}^{L}\right)}^{2}\right|}^{\gamma }+\xi_{8}{\left|{\left(\pi_{P_{C_{1}}}^{U}\right)}^{2}-{\left(\pi_{P_{C_{2}}}^{U}\right)}^{2}\right|}^{\gamma }+\xi_{9}{\left|{\left(\pi_{P_{C_{1}}}\right)}^{2}-{\left(\pi_{P_{C_{2}}}\right)}^{2}\right|}^{\gamma } \end{array}\right)\right)}^{\frac{1}{\gamma }}$$

where γ any positive integer.

If we consider multiple factors such as the MD, NMD, the strength of our commitment to a certain outcome, and the direction of commitment, while also taking into consideration the weight $\xi_{i}$ of each $t_{i}\in T$. we propose using the weighted distance measures $\delta_{\xi H}$ and $\delta_{\xi G}$ as follows:

(24) $$\delta_{\xi H}=\frac{1}{6}\left(\begin{array}{c} \xi_{1}\left|{\left(\varphi_{P_{C_{1}}}^{L}\right)}^{2}-{\left(\varphi_{P_{C_{2}}}^{L}\right)}^{2}\right|+\xi_{2}\left|{\left(\varphi_{P_{C_{1}}}^{U}\right)}^{2}-{\left(\varphi_{P_{C_{2}}}^{U}\right)}^{2}\right|+\xi_{3}\left|{\left(\psi_{P_{C_{1}}}^{L}\right)}^{2}-{\left(\psi_{P_{C_{2}}}^{L}\right)}^{2}\right|\\ +\;\;\xi_{4}\left|{\left(\psi_{P_{C_{1}}}^{U}\right)}^{2}-{\left(\psi_{P_{C_{1}}}^{U}\right)}^{2}\right|+\xi_{5}\left|{\left(\lambda_{P_{C_{1}}}\right)}^{2}-{\left(\lambda_{P_{C_{2}}}\right)}^{2}\right|\\ +\xi_{6}\left|{\left(\tau_{P_{C_{1}}}\right)}^{2}-{\left(\tau_{P_{C_{2}}}\right)}^{2}\right|+\;+\xi_{7}\left|{\left(\pi_{P_{C_{1}}}^{L}\right)}^{2}-{\left(\pi_{P_{C_{2}}}^{L}\right)}^{2}\right|+\xi_{8}\left|{\left(\pi_{P_{C_{1}}}^{U}\right)}^{2}-{\left(\pi_{P_{C_{2}}}^{U}\right)}^{2}\right|+\xi_{9}\left|{\left(\pi_{P_{C_{1}}}\right)}^{2}-{\left(\pi_{P_{C_{2}}}\right)}^{2}\right| \end{array}\right)$$

(25) $$\delta_{\xi G}={\left(\frac{1}{6}\left(\begin{array}{c} \xi_{1}{\left|{\left(\varphi_{P_{C_{1}}}^{L}\right)}^{2}-{\left(\varphi_{P_{C_{2}}}^{L}\right)}^{2}\right|}^{\gamma }+\xi_{2}{\left|{\left(\varphi_{P_{C_{1}}}^{U}\right)}^{2}-{\left(\varphi_{P_{C_{2}}}^{U}\right)}^{2}\right|}^{\gamma }+\;\xi_{3}{\left|{\left(\psi_{P_{C_{1}}}^{L}\right)}^{2}-{\left(\psi_{P_{C_{2}}}^{L}\right)}^{2}\right|}^{\gamma }\\ +\xi_{4}{\left|{\left(\psi_{P_{C_{1}}}^{U}\right)}^{2}-{\left(\psi_{P_{C_{1}}}^{U}\right)}^{2}\right|}^{\gamma }+\;\xi_{5}{\left|{\left(\lambda_{P_{C_{1}}}\right)}^{2}-{\left(\lambda_{P_{C_{2}}}\right)}^{2}\right|}^{\gamma }+\xi_{6}{\left|{\left(\tau_{P_{C_{1}}}\right)}^{2}-{\left(\tau_{P_{C_{2}}}\right)}^{2}\right|}^{\gamma }\\ +\;\xi_{7}{\left|{\left(\pi_{P_{C_{1}}}^{L}\right)}^{2}-{\left(\pi_{P_{C_{2}}}^{L}\right)}^{2}\right|}^{\gamma }+\xi_{8}{\left|{\left(\pi_{P_{C_{1}}}^{U}\right)}^{2}-{\left(\pi_{P_{C_{2}}}^{U}\right)}^{2}\right|}^{\gamma }+\;\xi_{9}{\left|{\left(\pi_{P_{C_{1}}}\right)}^{2}-{\left(\pi_{P_{C_{2}}}\right)}^{2}\right|}^{\gamma } \end{array}\right)\right)}^{\frac{1}{\gamma }}$$

**Theorem 1.** Let T be a non-empty set such that α,β,θ∈T. The distance measures are a metric δ on T that meets the following three properites:

a) δ(α,β)≥0, and δ(α,β)=0⇔α=β;
b) δ(α,β)=δ(β,α);
c) δ(α,β)=δ(α,θ)+δ(θ,β).

**Proof**. this proof is straightforward.

**Definition 20.** Let $P_{C_{1}}$ and $P_{C_{2}}$ be any two PCFNs on T=[a,b] and ξ be the weight vector, then normalized weighted Hamming distance (NWHD) between $P_{C_{1}}$ and $P_{C_{2}}$ can be defined as follows:

(26) $$\delta_{\xi H}\left(P_{C_{1}},P_{C_{2}}\right)=\frac{1}{6\left(b-a\right)}\int_{a}^{b}\left(\begin{array}{c} \xi_{1}\left|{\left(\varphi_{P_{C_{1}}}^{L}\right)}^{2}-{\left(\varphi_{P_{C_{2}}}^{L}\right)}^{2}\right|+\xi_{2}\left|{\left(\varphi_{P_{C_{1}}}^{U}\right)}^{2}-{\left(\varphi_{P_{C_{2}}}^{U}\right)}^{2}\right|+\xi_{3}\left|{\left(\psi_{P_{C_{1}}}^{L}\right)}^{2}-{\left(\psi_{P_{C_{2}}}^{L}\right)}^{2}\right|\\ +\;\xi_{4}\left|{\left(\psi_{P_{C_{1}}}^{U}\right)}^{2}-{\left(\psi_{P_{C_{1}}}^{U}\right)}^{2}\right|+\xi_{5}\left|{\left(\lambda_{P_{C_{1}}}\right)}^{2}-{\left(\lambda_{P_{C_{2}}}\right)}^{2}\right|+\xi_{6}\left|{\left(\tau_{P_{C_{1}}}\right)}^{2}-{\left(\tau_{P_{C_{2}}}\right)}^{2}\right|\\ +\xi_{7}\left|{\left(\pi_{P_{C_{1}}}^{L}\right)}^{2}-{\left(\pi_{P_{C_{2}}}^{L}\right)}^{2}\right|+\xi_{8}\left|{\left(\pi_{P_{C_{1}}}^{U}\right)}^{2}-{\left(\pi_{P_{C_{2}}}^{U}\right)}^{2}\right|+\xi_{9}\left|{\left(\pi_{P_{C_{1}}}\right)}^{2}-{\left(\pi_{P_{C_{2}}}\right)}^{2}\right| \end{array}\right)dt$$

**Definition 21.** Let $P_{C_{1}}$ and $P_{C_{2}}$ be any two PCFNs on T=[a,b] and ξ be the weight vector The NWED between $P_{C_{1}}$ and $P_{C_{2}}$ can be defined in the following way:

(27) $$\delta_{\xi E}\left(P_{C_{1}},P_{C_{2}}\right)=\frac{1}{6\left(b-a\right)}{\int_{a}^{b}\left(\begin{array}{c} \xi_{1}{\left|{\left(\varphi_{P_{C_{1}}}^{L}\right)}^{2}-{\left(\varphi_{P_{C_{2}}}^{L}\right)}^{2}\right|}^{2}+\xi_{2}{\left|{\left(\varphi_{P_{C_{1}}}^{U}\right)}^{2}-{\left(\varphi_{P_{C_{2}}}^{U}\right)}^{2}\right|}^{2}\;+\xi_{3}{\left|{\left(\psi_{P_{C_{1}}}^{L}\right)}^{2}-{\left(\psi_{P_{C_{2}}}^{L}\right)}^{2}\right|}^{2}\\ +\;\xi_{4}{\left|{\left(\psi_{P_{C_{1}}}^{U}\right)}^{2}-{\left(\psi_{P_{C_{1}}}^{U}\right)}^{2}\right|}^{2}\;+\xi_{5}{\left|{\left(\lambda_{P_{C_{1}}}\right)}^{2}-{\left(\lambda_{P_{C_{2}}}\right)}^{2}\right|}^{2}+\xi_{6}{\left|{\left(\tau_{P_{C_{1}}}\right)}^{2}-{\left(\tau_{P_{C_{2}}}\right)}^{2}\right|}^{2}\;\\ +\xi_{7}{\left|{\left(\pi_{P_{C_{1}}}^{L}\right)}^{2}-{\left(\pi_{P_{C_{2}}}^{L}\right)}^{2}\right|}^{2}+\xi_{8}{\left|{\left(\pi_{P_{C_{1}}}^{U}\right)}^{2}-{\left(\pi_{P_{C_{2}}}^{U}\right)}^{2}\right|}^{2}\;+\xi_{9}{\left|{\left(\pi_{P_{C_{1}}}\right)}^{2}-{\left(\pi_{P_{C_{2}}}\right)}^{2}\right|}^{2} \end{array}\right)}^{\frac{1}{2}}dt$$

**Definition 22.** Let $P_{C_{1}}$ and $P_{C_{2}}$ be any two PCFNs on T=[a,b] and ξ be the weight vector, then normalized generalized weighted distnace between $P_{C_{1}}$ and $P_{C_{2}}$ can be defined as follows:

(28) $$\delta_{\xi G}\left(P_{C_{1}},P_{C_{2}}\right)=\frac{1}{6\left(b-a\right)}\int_{a}^{b}{\left(\begin{array}{c} \xi_{1}{\left|{\left(\varphi_{P_{C_{1}}}^{L}\right)}^{2}-{\left(\varphi_{P_{C_{2}}}^{L}\right)}^{2}\right|}^{\gamma }+\xi_{2}{\left|{\left(\varphi_{P_{C_{1}}}^{U}\right)}^{2}-{\left(\varphi_{P_{C_{2}}}^{U}\right)}^{2}\right|}^{\gamma }\;+\xi_{3}{\left|{\left(\psi_{P_{C_{1}}}^{L}\right)}^{2}-{\left(\psi_{P_{C_{2}}}^{L}\right)}^{2}\right|}^{\gamma }\\ +\;\xi_{4}{\left|{\left(\psi_{P_{C_{1}}}^{U}\right)}^{2}-{\left(\psi_{P_{C_{1}}}^{U}\right)}^{2}\right|}^{\gamma }+\;\xi_{5}{\left|{\left(\lambda_{P_{C_{1}}}\right)}^{2}-{\left(\lambda_{P_{C_{2}}}\right)}^{2}\right|}^{\gamma }+\;\xi_{6}{\left|{\left(\tau_{P_{C_{1}}}\right)}^{2}-{\left(\tau_{P_{C_{2}}}\right)}^{2}\right|}^{\gamma }\\ +\xi_{7}{\left|{\left(\pi_{P_{C_{1}}}^{L}\right)}^{2}-{\left(\pi_{P_{C_{2}}}^{L}\right)}^{2}\right|}^{\gamma }+\xi_{8}{\left|{\left(\pi_{P_{C_{1}}}^{U}\right)}^{2}-{\left(\pi_{P_{C_{2}}}^{U}\right)}^{2}\right|}^{\gamma }\;+\xi_{9}{\left|{\left(\pi_{P_{C_{1}}}\right)}^{2}-{\left(\pi_{P_{C_{2}}}\right)}^{2}\right|}^{\gamma } \end{array}\right)}^{\frac{1}{\gamma }}dt$$

where γ is any positive integer.

## Application

Depression and anxiety are two common mental health disorders that affect millions of people worldwide. While they share some symptoms, such as feelings of sadness and hopelessness, they also have distinct characteristics. Different therapies for depression and anxiety may be evaluated and compared using multi-criteria decision-making (MCDM). This case study will examine the use of MCDM in mental health care decision making. How well does the therapy work to alleviate symptoms of sadness and anxiety? What are the risks associated with this treatment? Price, or how much money will you need to pay for this procedure. Accessibility refers to how easily one may get their hands on the necessary resources to undergo the therapy.

## Alternatives

1. Cognitive-behavioral therapy (CBT),
2. Selective serotonin reuptake inhibitors (SSRIs)
3. Mindfulness-based stress reduction (MBSR),
4. Exercise,
5. Dietary changes

### Methodology

**Fig. 1:**
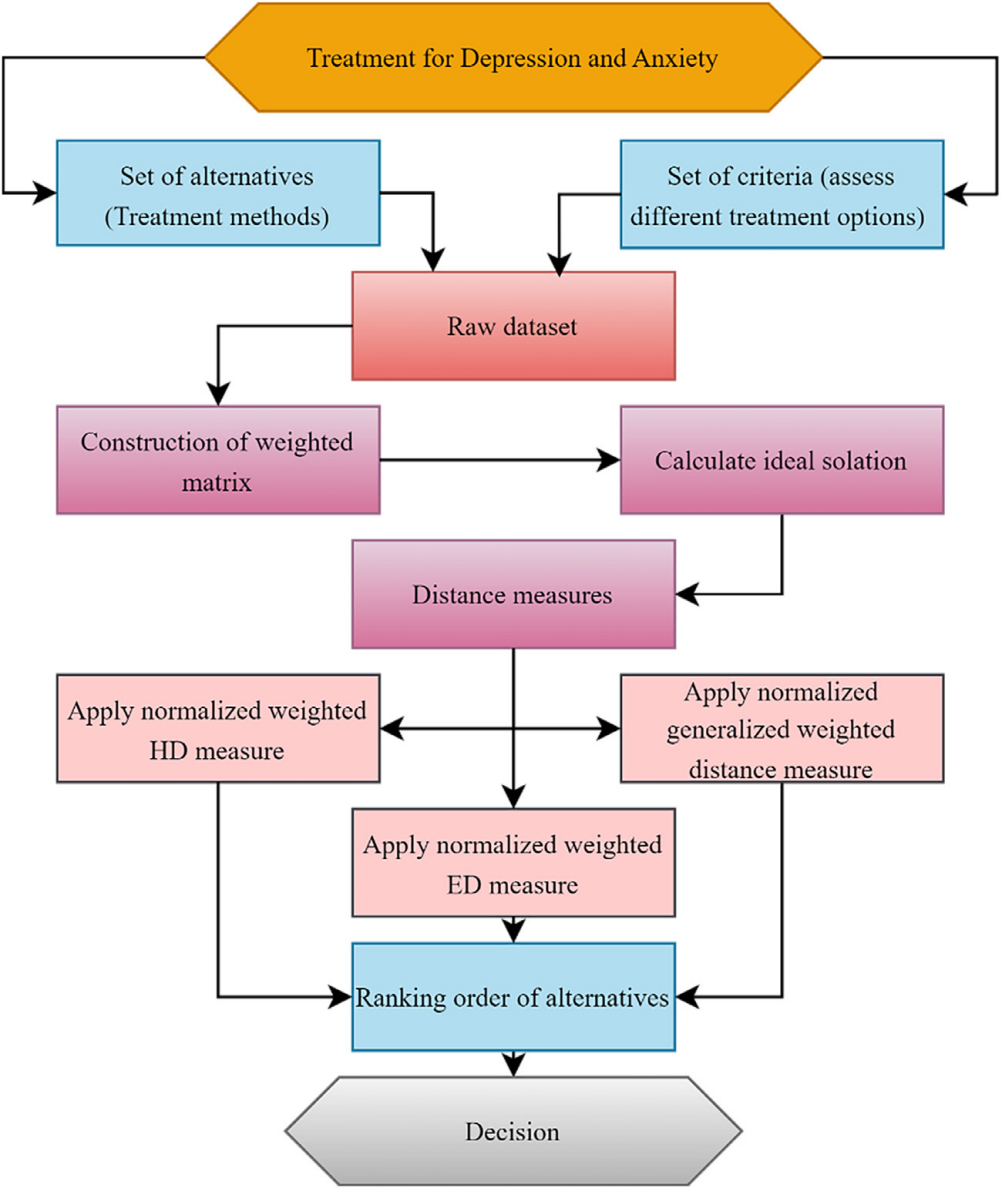
Proposed model.

In this research, we present PCFS-based distance measurements, which can provide a very flexible and modifiable framework for decision-makers to select the best possible option. With the help of these distance measurements, decision-makers may gain a more nuanced understanding of the trade-offs between available options by conducting a thorough examination of the interactions involving their selection criteria and the alternatives. By utilizing the PCFSs distance measures, decision makers can make more informed and effective decisions in complex decision-making scenarios. The notations $\zeta_{1}$, $\zeta_{2}$, $\zeta_{3}$, $\zeta_{4}$, and $\zeta_{5}$ represent CBT, SSIRs, MBSR, exercise, and dietary changes, respectively. All the components of our study are depicted in methodology framework illustrated in Fig. 1.

**Criteria 1.** Effectiveness of treatment $\eta_{1}$

**Table:** 

$\zeta_{1}$	(〈[0.7,0.8];0.6〉,〈[0.2,0.3];0.3〉)
$\zeta_{2}$	(〈[0.6,0.7];0.5〉,〈[0.4,0.5];0.3〉)
$\zeta_{3}$	(〈[0.4,0.6];0.5〉,〈[0.4,0.5];0.4〉)
$\zeta_{4}$	(〈[0.5,0.6];0.4〉,〈[0.4,0.5];0.3〉)
$\zeta_{5}$	(〈[0.2,0.3];0.2〉,〈[0.5,0.6];0.4〉)

**Criteria 2.** Side effects $\eta_{2}$

**Table:** 

$\zeta_{1}$	(〈[0.8,0.9];0.7〉,〈[0.1,0.2];0.2〉)
$\zeta_{2}$	(〈[0.5,0.6];0.6〉,〈[0.3,0.4];0.4〉)
$\zeta_{3}$	(〈[0.8,0.9];0.8〉,〈[0.1,0.3];0.3〉)
$\zeta_{4}$	(〈[0.8,0.9];0.9〉,〈[0.1,0.2];0.4〉)
$\zeta_{5}$	(〈[0.8,0.9];0.8〉,〈[0.1,0.3];0.3〉)

**Criteria 3.** Affordability $\eta_{3}$

**Table:** 

$\zeta_{1}$	(〈[0.3,0.4];0.6〉,〈[0.6,0.7];0.2〉)
$\zeta_{2}$	(〈[0.6,0.7];0.5〉,〈[0.3,0.4];0.7〉)
$\zeta_{3}$	(〈[0.2,0.3];0.8〉,〈[0.6,0.7];0.5〉)
$\zeta_{4}$	(〈[0.8,0.9];0.8〉,〈[0.1,0.2];0.4〉)
$\zeta_{5}$	(〈[0.8,0.9];0.3〉,〈[0.4,0.5];0.8〉)

**Criteria 4.** Accessibility $\eta_{4}$

**Table:** 

$\zeta_{1}$	(〈[0.5,0.6];0.3〉,〈[0.4,0.5];0.7〉)
$\zeta_{2}$	(〈[0.7,0.8];0.4〉,〈[0.4,0.5];0.8〉)
$\zeta_{3}$	(〈[0.3,0.4];0.8〉,〈[0.6,0.7];0.5〉)
$\zeta_{4}$	(〈[0.7,0.8];0.7〉,〈[0.3,0.4];0.4〉)
$\zeta_{5}$	(〈[0.6,0.7];0.4〉,〈[0.3,0.4];0.6〉)

After weighting each criterion according to its relative importance, we can calculate the overall scores for each alternative. Assuming that the most important criterion is effectiveness (0.4 weight), followed by side effects (0.25 weight), affordability (0.2 weight), and accessibility (0.15 weight), the overall scores for each alternative are as follows:

Eq. (14) is used to calculate the Pythagorean cubic fuzzy ideal solution. The positive ideal solutions are summarized in Table 1.

**Table 1:** Positive ideal solutions.

(〈[0.6,0.8];0.5〉,〈[0.6,0.7];0.4〉)
(〈[0.6,0.8];0.5〉,〈[0.4,0.8];0.7〉)
(〈[0.6,0.7];0.4〉,〈[0.5,0.6];0.2〉)
(〈[0.6,0.8];0.5〉,〈[0.6,0.7];0.4〉)
(〈[0.6,0.8];0.5〉,〈[0.6,0.7];0.4〉)

Using Eq. (19) to calculate the NHWD between $\zeta_{i}$ and $\zeta^{+}$. Distance measures and ranking of alternatives shown in Table 2.

**Table 2:** NWHD measures and ranking order of alternatives.

Approach	$\zeta_{1}$	$\zeta_{2}$	$\zeta_{3}$	$\zeta_{4}$	$\zeta_{5}$
$\delta_{\xi H}\left(\zeta_{i},\zeta^{+}\right)$	0.0831	0.0512	0.0263	0.0649	0.0476
ranking	1	3	5	2	4

Using Eq. (22) to determine the NED measures between $\zeta_{i}$ and $\zeta^{+}$. Table 3 represents the NED measures and ranking of alternatives.

**Table 3:** Normalized weighted Euclidean distance measures and ranking of alternatives.

Approach	$\zeta_{1}$	$\zeta_{2}$	$\zeta_{3}$	$\zeta_{4}$	$\zeta_{5}$
$\delta_{\xi H}\left(\zeta_{i},\zeta^{+}\right)$	0.1250	0.0928	0.0574	0.1082	0.0871
ranking	1	3	5	2	4

Finally, using Eq. (25) to calculate the normalized weighted generalized distance measures between $\zeta_{i}$ and $\zeta^{+}$ for γ=2. The results are summerized in Table 4.

**Table 4:** Normalized generalized weighted distance measures and ranking of alternatives.

Approach	$\zeta_{1}$	$\zeta_{2}$	$\zeta_{3}$	$\zeta_{4}$	$\zeta_{5}$
$\delta_{\xi H}\left(\zeta_{i},\zeta^{+}\right)$	0.1489	0.1015	0.0890	0.1216	0.0954
ranking	1	3	5	2	4

**Fig. 2:**
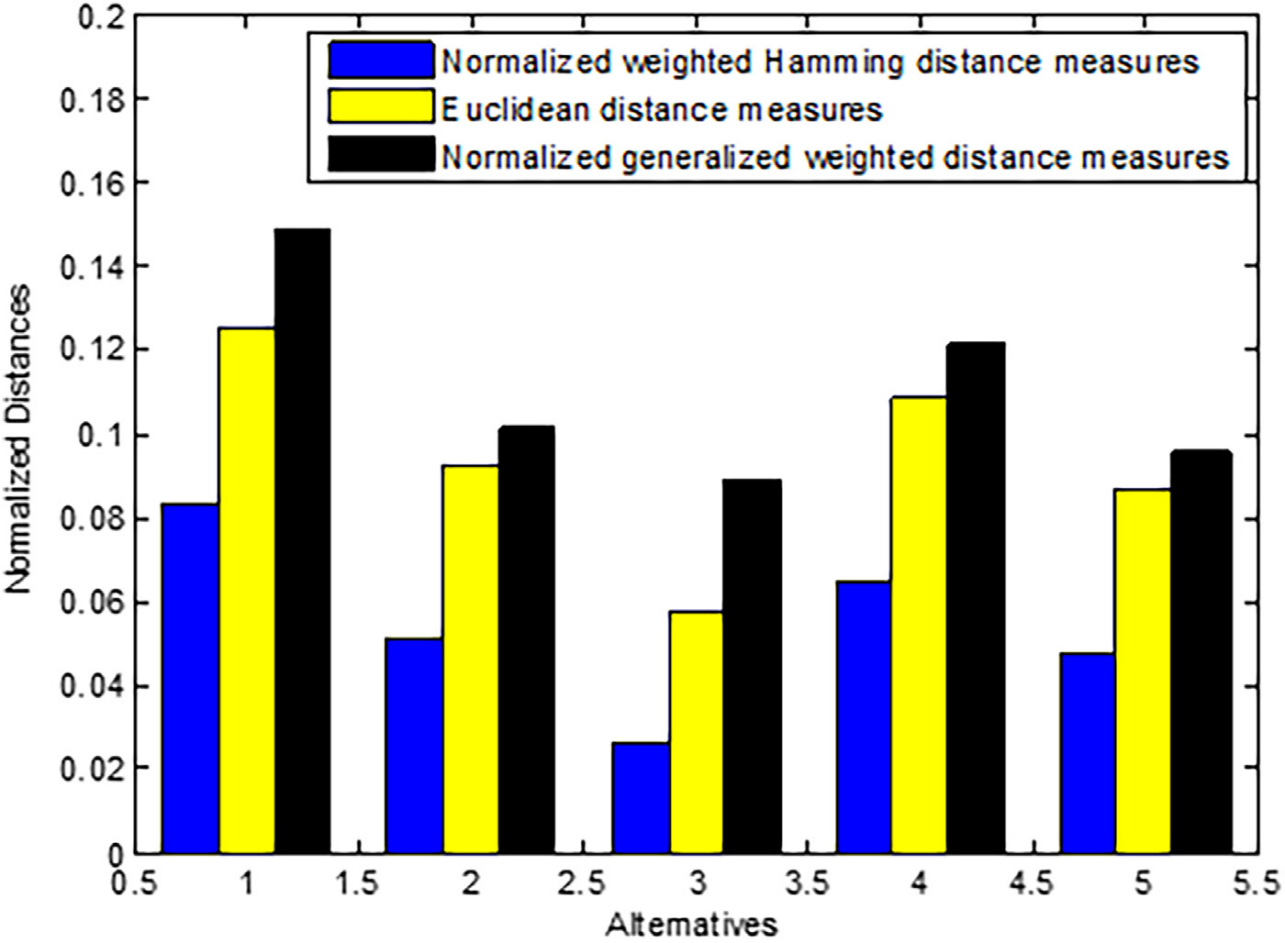
Different normalized distance metrics and their differences are graphically depicted.

Based on the results of our MCDM analysis, CBT ($\zeta_{1}$) and exercise ($\zeta_{4}$) are the most promising treatment options for depression and anxiety, with high scores for effectiveness and accessibility. While SSRIs are effective, they have some potential side effects and may not be as accessible or affordable as other options. Although mindfulness-based stress reduction (MBSR) and dietary adjustments do have some positive effects, they are not as effective or cost-efficient as other options. In sum, MCDM is a helpful method for analyzing and contrasting various therapies for mental health problems. Fig. 2 is a graphical representation of the distance metrics that were proposed.

### Comparative study

We've compared our suggested distance metrics to industry standards to get a full representation of how well they work. Our hope is that this comparison will show how much better our new metrics are. The details of the distance metrics that have to compare with are summarized in Table 5 for ease of comprehension. Table 5 shows that compared to other methods that use general Pythagorean fuzzy sets like IVPFSs or PFSs, our suggested decision-making methodology using cubic Pythagorean fuzzy sets is more successful. The justification for this is because our suggested technique incorporates both PFS and IVPFS data into the evaluation of alternatives, whereas existing methods only incorporate PFS data. As a result, the information about the alternatives may be missed by the distance measurements utilized in the present methodologies, leading to less reliable choice outcomes.

**Table 5:** Methods currently used and a ranking to other options.

Current distance methods	alternatives					Ranking
	$\zeta_{1}$	$\zeta_{2}$	$\zeta_{3}$	$\zeta_{4}$	$\zeta_{5}$	
Li and Zeng [38]	0.0701	0.0589	0.0137	0.0611	0.0382	$\zeta_{1}≻\zeta_{4}≻\zeta_{2}≻\zeta_{5}≻\zeta_{3}$
Wang and Xin [39]	0.0932	0.0744	0.0236	0.0907	0.0456	$\zeta_{1}≻\zeta_{4}≻\zeta_{2}≻\zeta_{5}≻\zeta_{3}$
Zeng [40]	0.0747	0.0626	0.0302	0.0719	0.0390	$\zeta_{1}≻\zeta_{4}≻\zeta_{2}≻\zeta_{5}≻\zeta_{3}$
He et al. [41]	0.0325	0.0104	0.0015	0.0201	0.0086	$\zeta_{1}≻\zeta_{4}≻\zeta_{2}≻\zeta_{5}≻\zeta_{3}$

**Fig. 3:**
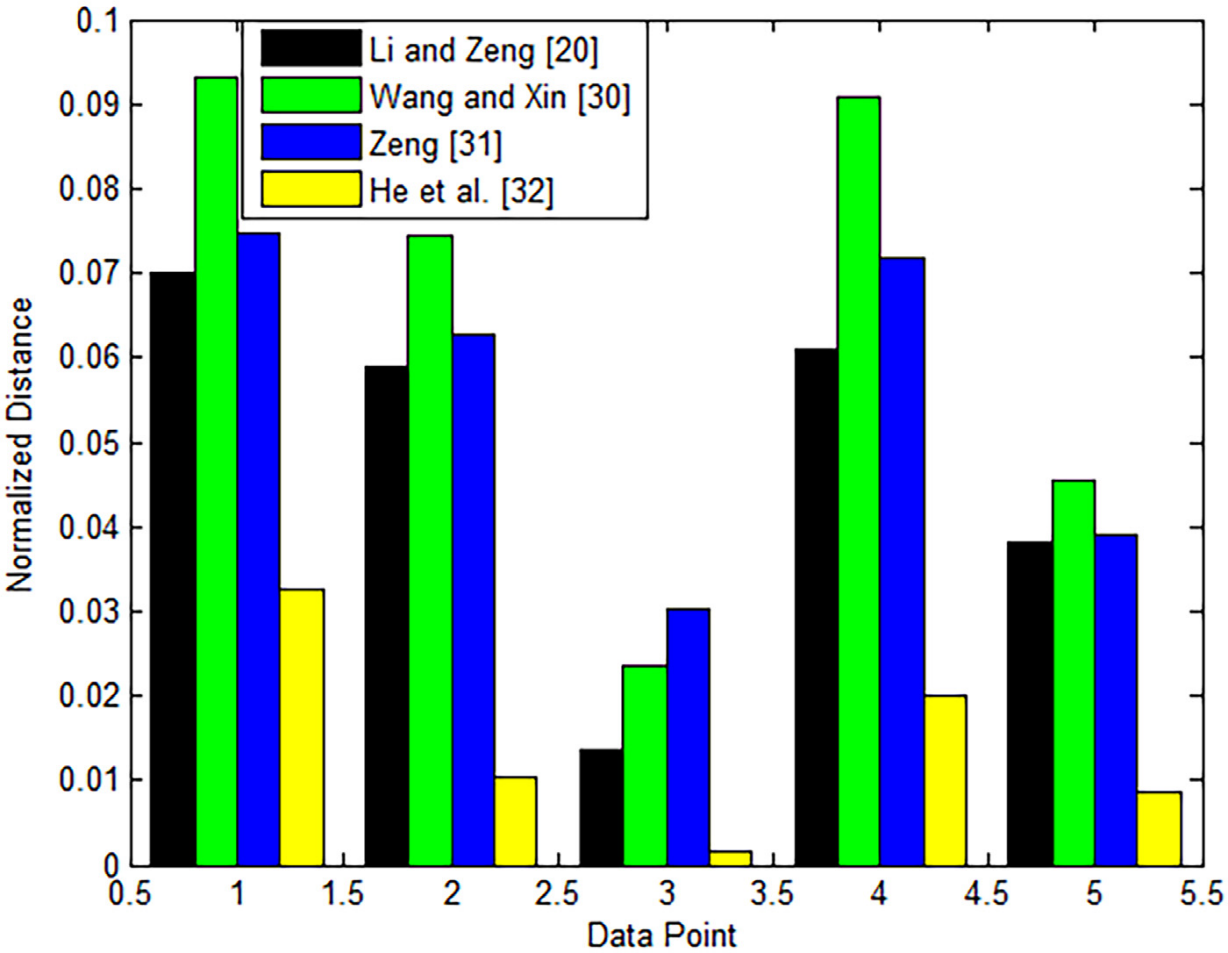
the visual representation of the alternative, showing how several methods now in use compare.

In conclusion, the results of our analysis show that our suggested distance measures are more accurate than the already used measures, showing the potential advantages of applying cubic Pythagorean fuzzy sets in decision-making processes. Table 5's graphical representation may be seen in Fig. 3.

### Results and discussions

The MM degrees of Pythagorean cubic fuzzy sets are determined by the values of Pythagorean triples, making this a special case of FSs based on the Pythagorean theorem. Similarity to the Pythagorean triple is used to calculate the MM grade of a value in this fuzzy set. Such collections are employed in several contexts, including decision-making and pattern identification. They can be used alone, or in conjunction with other FSs like the triangular and trapezoidal ones, to build more sophisticated and precise models. They can be helpful in many areas by offering a more practical, adaptable perspective.

The research found that the suggested distance measures employ a unique computational approach, unlike the distance measures used in previous situations. In the appraisal process, these recommended arrangements are seen as more acceptable and feasible. When both the upper and lower limits of membership degrees are equal, the set of measures utilized in past studies [42,43] and [44] can be viewed as a special case of the suggested distance measures. Thus, the suggested method is more universal and applicable to a broader range of circumstances since it is more thorough and allows for additional information during the research. A more practical and adaptable method of dealing with uncertainty, PCFSs may be used to a variety of decision-making challenges. The outcomes show a considerable improvement in the quality of decisions made and the capacity for accurately modeling difficult choice issues. These results show that Pythagorean cubic fuzzy sets have promise for improving decision support systems in a wide range of fields, and they open the door to more study in this area.

### Sensitivity analysis

The suggested distance measures were used to compare different solutions and their rankings, considering the range and sensitivities of the parameter. Table 6 provides a summary of the findings. varied values of the parameter in the suggested distance measurements provide varied results, as shown in Table 6. The relative rankings of the options for the different values of are unaffected by the disparities in result values. It is also shown that when the parameter grows, the result values drop. It follows that when is between 2 and 6, decision-makers are more likely to take a positive stance, whereas a bigger value of indicates a more pessimistic disposition. It's worth noting that various authorities may decide to use varying values for based on their own needs and preferences. Therefore, experts' choices about the parameter may differ according to their own requirements and worldviews.

**Table 6:** Ranking order of alternatives for different values of γ.

γ	$\zeta_{1}$	$\zeta_{2}$	$\zeta_{3}$	$\zeta_{4}$	$\zeta_{5}$	Ranking order
2	0.1250	0.0928	0.0574	0.1082	0.0871	$\zeta_{1}≻\zeta_{4}≻\zeta_{2}≻\zeta_{5}≻\zeta_{3}$
3	0.1236	0.0917	0.0567	0.1071	0.0867	$\zeta_{1}≻\zeta_{4}≻\zeta_{2}≻\zeta_{5}≻\zeta_{3}$
4	0.1223	0.0911	0.0559	0.1065	0.0863	$\zeta_{1}≻\zeta_{4}≻\zeta_{2}≻\zeta_{5}≻\zeta_{3}$
5	0.1207	0.0902	0.0551	0.1060	0.0858	$\zeta_{1}≻\zeta_{4}≻\zeta_{2}≻\zeta_{5}≻\zeta_{3}$
6	0.1185	0.0892	0.0547	0.1058	0.0851	$\zeta_{1}≻\zeta_{4}≻\zeta_{2}≻\zeta_{5}≻\zeta_{3}$

By referring to Fig. 3, we observe that the proposed method and the existing approach exhibit the same ranking order for alternatives. It's important to keep in mind that the current methods are all developed for either an intuitionistic fuzzy or a Pythagorean fuzzy setting. The limitation of these existing methods lies in the fact that decision-makers are unable to provide rating values for the alternatives. Consequently, the proposed distance measures offer a more flexible environment for addressing complex decision-making problem.

**Fig. 4:**
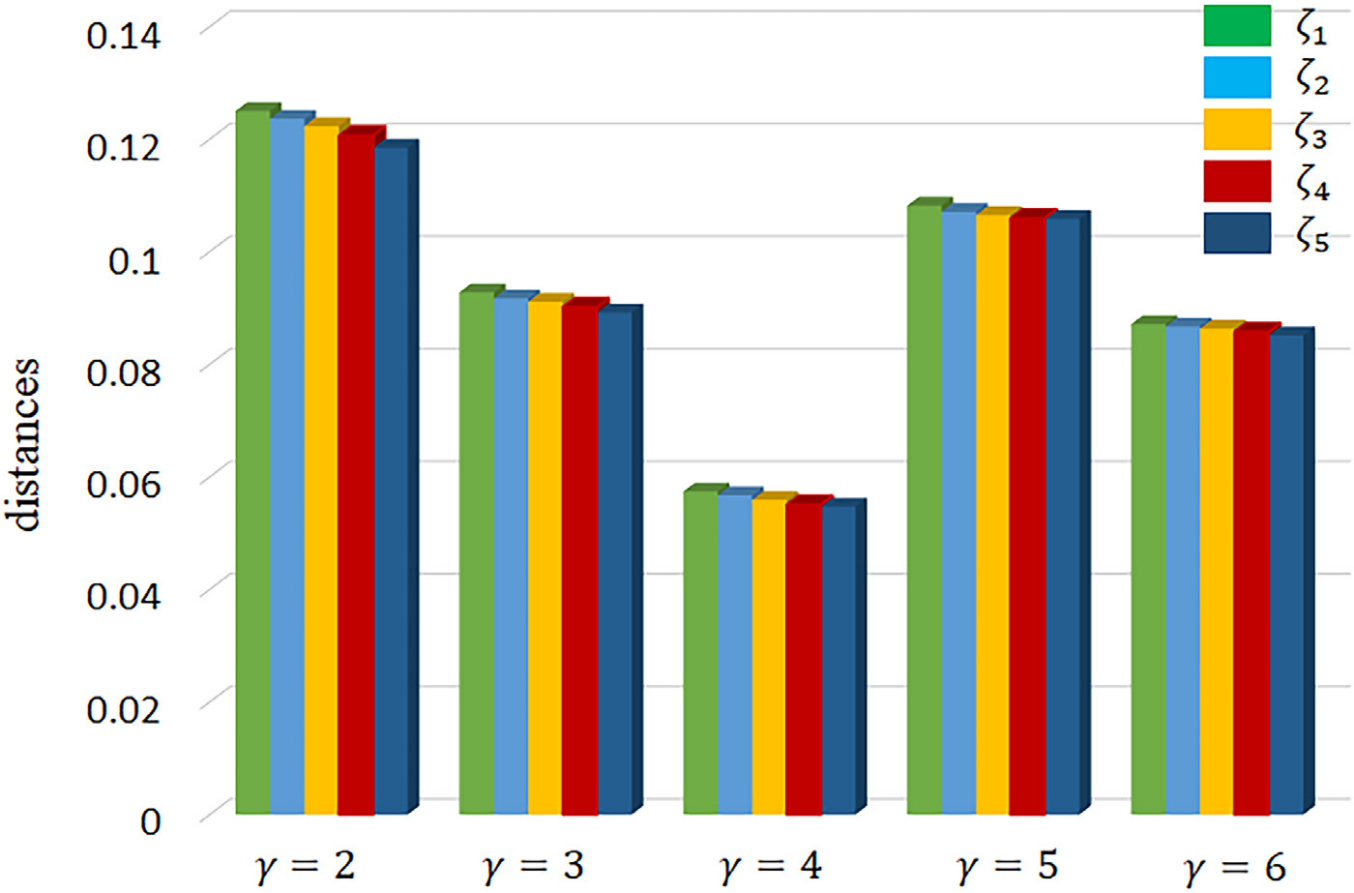
The Ranking of alternatives for different γ.

By Fig. 4, it is apparent that an increase in the parameter γ value leads to a gradual decrease in the distance between two CPFNs. This observation suggests that decision makers have the flexibility to assign lower values to this parameter γ during the decision-making process if they possess an optimistic outlook. Conversely, if decision makers lean towards pessimism, higher values can be assigned to parameter γ resulting in higher score values for the overall evaluation. However, it is important to note that regardless of the decision makers' optimism or pessimism, the best alternative remains the same. This finding underscores the objectivity of the results, which are not influenced by decision makers' preferences for pessimism or optimism. Consequently, the range of values for parameter γ may vary based on decision makers' viewpoint, but the ultimate outcome remains unaffected.

Our proposed tool has the following advantages:

1. By utilizing PCFSs and DMs of PCFSs, we can overcome the limitations of traditional fuzzy sets and develop more operative techniques for decision-making. Our proposed distance measures offer a more comprehensive and accurate approach that can capture the intricate relationships between different elements of CPFSs.
2. We showcase the effectiveness of our method by providing an example in the context of material treatment. The example highlights how our proposed distance measures excel in managing PCFSs compared to traditional decision-making techniques, demonstrating the superiority of our approach. The example clearly demonstrates how our method can deliver more precise outcomes and facilitate better decision-making across different situations.

Like any other decision-making tool, the MCDM also has its limitations. Some of the limitations of MCDM are:

1. In the proposed decision-making process, we evaluated four criteria and five alternatives. However, there is room for improvement in the results by incorporating additional criteria and alternatives, which could be explored by future scholars.
2. The results of proposed MCDM can be sensitive to the aggregation method used, i.e., different aggregation methods may lead to different results.
3. The proposed MCDM assumes that the criteria used in the analysis are independent of each other, but criteria may be interdependent.

## Conclusion

In the realm of Pythagorean cubic fuzziness, it is crucial to identify suitable distance measures for assessing the divergence between Pythagorean cubic fuzzy sets. Such measures are fundamental for devising distance-based compromise methods within the Pythagorean cubic fuzzy framework. However, the current distance measures employed in the Pythagorean cubic fuzzy theory present several limitations and challenges. To tackle this issue, this paper presents novel distance measures specifically tailored for Pythagorean cubic fuzziness, which serve as a fundamental technique for handling intricate and sophisticated information that employs Pythagorean cubic membership grades to accommodate the intricate uncertainties of MCDM issues. The new distance methods are suggested as a solution to the constraints and difficulties encountered with the existing Pythagorean cubic fuzzy distance measures and are expected to furnish more precise and dependable outcomes in the scrutiny of ambiguous data in MCDM. A tangible example is delivered to demonstrate the effectiveness of the projected MCDM methodology in selecting the optimal treatment alternative for depression and anxiety.

In the future, it would be advantageous to extend the distance measure anticipated in this paper to the realm of medical diagnosis, for use in the development of fuzzy decision-making problems. Moreover, the proposed distance measure can be expanded as a future study to encompass linguistic Pythagorean cubic fuzzy sets or linguistic cubic intuitionistic fuzzy sets. Such an extension would allow the distance measure to handle more intricate and sophisticated information involving linguistic terms, which are frequently employed in medical diagnosis. This would be a significant stride towards utilizing the proposed measure in practical situations and enhancing the accuracy of the decision-making process in the medical domain.

## Ethics statements

The authors did not conduct any studies involving human or animal participants for this article.

## CRediT authorship contribution statement

**Muhammad Rahim:** Conceptualization, Methodology. **Fazli Amin:** Validation, Data curation, Writing – original draft. **Kamal Shah:** Visualization, Investigation. **Thabet Abdeljawad:** Supervision, Software, Validation. **Sadique Ahmad:** Writing – original draft, Investigation, Software.

## Declaration of competing interest

The authors declare that they have no known competing financial interests or personal relationships that could have appeared to influence the work reported in this paper.

## Data Availability

This article does not require data sharing as no datasets were used or generated during the current study.
